# Amyloidogenic Growth Observation of Stem Bromelain via Atomic Force Microscopy

**DOI:** 10.1021/acsomega.5c07595

**Published:** 2025-10-09

**Authors:** Maria Christine Lugo, Atsushi Kammura, Toshiharu Kobayashi, Masahiro Ito, Takunori Harada, Kazuo Umemura

**Affiliations:** † Department of Physics, 26413 Tokyo University of Science, 1-3 Kagurazaka, Shinjuku, Tokyo 162-8601, Japan; ‡ Department of Medical Course, Teikyo Heisei University, 2-51-4 Higashi-ikebukuro, Toshima, Tokyo 1708445, Japan; § Department of Integrated Science and Technology, Faculty of Science and Technology, Oita University, 700 Dannoharu, Oita City 870-1192, Japan

## Abstract

In this paper, we report on the amyloidogenic fibril formation of stem bromelain (SB) by using atomic force microscopy (AFM). Stem bromelain (SB), a proteolytic enzyme, is widely used in industries and medicine, making it essential to understand the factors affecting aggregation. Amyloid formation entails the assembly of proteins into highly ordered, β-sheet-rich fibrillar structures; yet while heating is a recognized trigger for SB fibrillation, the extent of continued fibril growth at room temperature incubation and its nanoscopic morphological observation remain unexplored. Here, SB was heated in pH 10.8 borate buffer at 65 °C for 10 h, then incubated at room temperature for 1, 3, and 7 days, respectively. A time-course imaging directly visualized the morphological progression from small, dispersed protofibrils on day 1 to increasingly pronounced fibrillar bundles on day 3 and dense, interconnected amyloid networks by day 7. Quantitative analysis of AFM images revealed a progressive increase in alignment in the orientation distribution, which shows directional growth of fibril on mica substrate. Moreover, there is a clear upward trend in fibril coverage area over time, with day 7 showing significantly higher coverage, which implies structural organization. We also introduce a technique that provides an accessible, high-resolution approach for real-time morphological studies of SB protofibril elongation and provides new insights into the kinetics and organizational dynamics of amyloid fibril formation.

## Graphical abstract

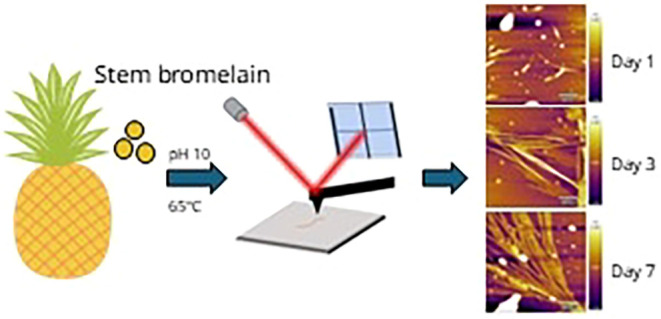

## Introduction

Atomic force microscopy (AFM) is a powerful biophysical tool used in the study of atomic to nanoscopic characteristics of biomaterials^1–5^ offering nanoscale resolution and the ability to monitor morphological changes in biomaterials like protein assemblies. It operates by employing a sharp tip mounted on a cantilever that physically scans the surface of the sample^6^ both in liquid and in air. As the tip encounters changes in surface height, it deflects in response to interatomic forces, and these deflections are measured, producing high-resolution images of the topography. This precision allows researchers to visualize single molecules, aggregates, and the intricate architecture of biomaterials in exceptional detail. Among the biomolecular structures that can be studied using AFM are protein assemblies like amyloid fibrils, although these are more commonly investigated using spectroscopic or staining techniques.^7–10^ Notably, AFM offers the advantage of observing biological samples in their native state.^7,11^ Amyloid fibrils originate from naturally soluble proteins that assemble into highly ordered, insoluble structures resistant to degradation.^7,12–15^ Furthermore, recent AFM results have shown that oligomeric fibril aggregation is not necessarily true amyloids like the worm-like fibril formation of S100A9 protein.^16^ While spectroscopic and diffraction techniques provide ensemble-averaged information about the internal molecular structure of fibrils, averaging limits their ability to explain the variations in the height, width, structure, and real-time growth, which is evident with imaging using AFM.

Amyloid fibrils gained much attention because of their association with various diseases like neurodegenerative Alzheimer’s disease, amyotrophic lateral sclerosis (ALS), and type II diabetes, all of which are progressive disorders with high morbidity.^13,17–19^ However, researchers then found that it is widespread and that proteins unrelated to disease can form highly stable nanostructures with distinctive functional roles. Functional amyloids have been identified from bacteria to humans^20^ like human Pmel17 for its role in the biosynthesis of melanin.^21^ Recent advances have exploited its potential application in medicine, material science, engineering, and sensors.^14,22–24^ Progressive studies have utilized nontoxic amyloid fibrils for cell adhesion and tissue engineering applications due to its extracellular matrices’ mimetic surface topography and ability to mediate active cell adhesion.^22^ The biocompatibility and structural stability of amyloid fibril-based biomaterials open avenues for sustainable alternatives to the conventionally used synthetic materials.

Another protein that is extracted from the stems of pineapple (*Ananas comosus*) has been identified to be capable of forming amyloid fibrils under specific conditions.^25,26^ Stem bromelain (SB) is a cysteine protease widely accepted as a potential phototherapeutic drug due to its broad medicinal applications^27^ such as reversible inhibition of platelet aggregation and enhanced adsorption of drugs, especially antibiotics. Like other cysteine proteases, SB belongs to α + β protein class and has a wide variety of effective enzyme activities at both acidic and alkaline conditions.^28–30^ Previous studies suggest that SB undergoes amyloid fibrillation under specific conditions, such as heat treatment or the presence of surfactants. SB, when heated at pH 10, showed amyloidogenic growth as confirmed through binding to indicative dyes, inherent luminescence, and increase in hydrodynamic radii.^25^

In this study, we use AFM to investigate the amyloid fibrillation behavior of stem bromelain (SB) by examining the effects of incubation at room temperature following heat treatment. We observed the extended growth of SB amyloid fibrils and analyzed the localized spatial organization and alignment of the fibrils on mica substrates, providing insight into their orientational growth over time. This AFM study of SB highlights a novel observation that has not been previously reported by using other macromolecular techniques. Additionally, we visualized the morphological progression of SB from early oligomeric forms to protofibrillar structures. This work contributes to a deeper understanding of SB fibril formation dynamics and offers a foundation for potential applications in functional biomaterials or industrial protein design. Given SB’s established use in medicine and industry, our findings open avenues for its application as a biobased material such as designing biocompatible scaffolds or nanostructured enzyme platforms.

## Materials and Methods

### Materials and Sample Preparation

Stem bromelain (SB) (from *A. comosus* B4882) was purchased from Sigma-Aldrich Chemical Co. (MO) and was used as received. Borate buffer solution of pH 10.8 (100 mM) and sodium phosphate buffer solution of pH 7.0 (10 mM) were prepared. The pH measurements were carried out using a Horiba (Laqua F-71) pH meter. Prior to use, buffer solutions were filtered by using PVDF 0.45 μm syringe filters (Millipore Milex-HV). SB was dissolved in sodium phosphate buffer (10 mM) with a concentration of 0.5 mg/mL. Also, SB was dissolved in borate buffer (100 mM) with a concentration of 0.5 mg/mL and incubated at 65 °C for 10 h. The rationale of the heating temperature and incubation time was based on a previous study by Zaman et al.^25^ The pH measurements were also checked post-dissolution of SB and were found to have no significant difference in the pH of solution.

### Atomic Force Microscopy

To deposit SB solutions on the mica substrate for time-course observation, 10 μL of sample was placed on parafilm and covered with a bare mica substrate for 10 min. The substrate was then washed with 100 μL of ultrapure water, 3 times. The substrate was then incubated inside a Petri dish where humidity was kept constant by putting droplets of water and sealing it for 1, 3, and 7 days, respectively. This will increase local humidity and slow the evaporation. This helps to prevent the influence of drying artifacts on fibril growth. Then, the samples were vacuum-dried before AFM observations for consistent sample condition.

For real-time observation of SB fibrillation, 10 μL of solution was dropped on parafilm, covered by bare mica for 10 min, and washed 3 times with 100 μL of ultrapure water. It was then incubated for 1 day at constant humidity and vacuum-dried for up to 1 h. Maintaining a moist environment for the bare mica is essential for observing amyloidogenic growth by using AFM.

The prepared substrate was then attached to the sample holder by using double-sided tape. AFM imaging in air was performed in AC mode using an MFP-3D microscope (Asylum Research, CA) with a scan rate of 1 Hz and a scan time of 4 min and 16 s. A silicon nitride microcantilever (BL-AC40TS-C2, Olympus, Japan) with a nominal spring constant of 0.09 N/m was used. AFM calibration was checked using a platinum-coated calibration grid (Digital Instruments, Veeco Metrology group) with 1 μm × 1 μm period, as seen in Figure S9. Height profile (Figure S9c) was obtained along a line scan across multiple grids to confirm length accuracy. The measured lateral spacing was 1 μm, which is consistent with the manufacturer’s specifications, confirming accurate calibration in the lateral dimensions. Three cantilevers were used to check the calibration.

An initial investigation was also done to observe SB in AFM in a liquid environment. Ten microliters of prepared sample solution for pH 7 and pH 10.8 were dropped on a parafilm covered by mica substrate for 10 min and then washed with 100 μL of ultrapure water three times. The prepared substrates were glued to the bottom of a closed fluid cell (CFC) (939.010, Asylum Research, CA) and soaked in 2000 μL of buffer solution. AFM in liquid (AC mode, MFP-3D, Asylum Research, CA) measurement was conducted at room temperature. However, liquid measurement was not successful at pH 10.8 due to the electrostatic repulsion of SB fibrils (positively charged) to a negatively charged mica substrate. Silanization on mica substrate using (3-aminopropyl) triethoxysilane (APTES)-glutaraldehyde was also performed to prevent electrostatic repulsion; however, due to strong bonding, it hinders the growth of SB fibrils on the substrate. The experimental method for deposition on APTES–glutaraldehyde-treated mica is described in Figure S6.

### Circular Dichroism (CD) Measurements

Circular dichroism (CD) and absorption spectra of samples (0.5 mg/mL) were measured on a CD spectrophotometer (JASCO: J-1500) in the borate buffer solution (pH 10.8) at room temperature to compare the unheated stem bromelain solution to the heated and post-incubated for 7 days stem bromelain solution. The spectra were recorded over a wavelength range of 198–260 nm with “Standard” sensitivity at 50 nm/min with 1 nm resolution, time constant of 1 s. Data were further processed for noise reduction if necessary. The CD and absorption signals were presented as ellipticities (mdeg) and as optical density (OD).

Percentages of the protein secondary structure motifs were estimated with the BeStSel software, developed by Kardos et al.^31^ The BeStSel web server is freely accessible at http://bestsel.elte.hu.

### Coverage Area Analysis

The phase retrace of AFM images was processed in ImageJ to quantify the coverage area of stem bromelain fibrils. The images were first cropped to remove the outer frame associated with AFM scans and then converted to 8-bit grayscale. Threshold was performed using ImageJ’s “Threshold” function and set to B&W (black and white) to generate binary images, where fibrils appeared white against a black background. Threshold adjustments were set to around 125 for lower threshold level and 255 for upper threshold level, to isolate brighter features (like fibrils) and remove the dimmer background. Thirty images of 5–10 μm AFM scans selected and analyzed for each incubation time. The fibril coverage was then quantified by calculating the percentage area (%area) using the formula

1 $$\%\mathrm{area}=\frac{\mathrm{total}\;\mathrm{fibril}\;\mathrm{area}\;\left(\mathrm{pixels}\right)}{\mathrm{total}\;\mathrm{area}\;\left(\mathrm{pixels}\right)}\times 100\%$$

This measurement provided an estimate of the fibril distribution within the analyzed region. All retrieved data were presented as the mean ± standard deviation, obtained from analysis of 30 AFM images collected across three independent experiments for each condition. To compare the mean values of the fibril distribution depending on the days of incubation *t*-test statistical analysis was done. Comparisons were conducted between days 1 and 3, days 1 and 7, and days 3 and 7, respectively. Accordingly, the *p*-value for the statistical difference was set to be 0.05.

### Orientational Analysis

To quantitatively analyze fibril orientation, the MATLAB FiberApp application was utilized.^32^ Each fibril’s initial position was manually defined with the fitting calculated using the A* path-finding algorithm which is embedded within the software.^33^ AFM images with resolution of 2125 × 1840 pixels were used to extract data from fibrils. Using the software, polar histograms and order parameter (*S*_2D_) are generated from individual AFM images with angular measurements binned in 5° increments. The value *S*_2D_ is defined by the equation

2 $$S_{2D}=2\left\langle \cos^{2}\left(\theta_{n}\right)\right\rangle -1$$

where θ is the angle between the *n*th segment and the local director in the chosen area. This metric quantifies the degree of orientational alignment, with higher *S*_2D_ values indicating greater alignment.

The normalized orientation distribution of stem bromelain fibrils was obtained by first processing the angular data such that the peak orientation was aligned to 0°, enabling a standardized comparison across all samples. Orientation measurements were extracted from 30 AFM images across 3 independent experiments per condition, with scan sizes ranging from 10 to 20 μm. For each condition, the mean and standard deviation of the fibril angles were calculated. The angular data were subsequently normalized by setting the intensity at 0° to unity, allowing for direct, quantitative comparison of orientation distributions across different time points.

## Results and Discussion

Stem Bromelain (SB) fibril has an isoelectric point of 9.5, which has a net negative charge at pH 10.8. Attachment of SB fibril in bare mica substrate while suspended in liquid was not possible due to electrostatic repulsion. Initial experiment conducted in liquid (see Figure S5a,b) showed the detachment of SB particles on the mica substrate, while in Figure S5c,d, the APTES (3-aminopropyl triethoxysilane) glutaraldehyde substrate allowed the attachment of SB. However, the effect of strong attachment made the fibrillation of SB on APTES-glutaraldehyde mica unsuccessful. We opted to perform all native bromelain and fibril growth observation on bare mica substrate in an air environment. In Figure 1a, the stem bromelain remains in its native, stable conformation at pH 7 appearing as discrete, rounded particles, indicative of its structural stability. In contrast, at pH 10.8 (Figure 1b), the protein undergoes aggregation, forming larger structures. This is attributed to increased hydrophobic interaction and partial unfolding induced by the alkaline environment. Previous studies have demonstrated that SB undergoes conformational change near its isoelectric point (pI around 9.5).^25,26,28,29^ At basic pH (10.0), stem bromelain undergoes structural changes where its hydrophobic core becomes more compact and exposed. Although most of its secondary structure is retained, the loss of tertiary interactions suggests that SB adopts a molten globule state under these conditions.^25,29^ Due to the nature of protein in dried environments, probable flattening effects and structural change might occur during the drying process of AFM in air observation. AFM in liquid images is provided in Supporting Information Figures S5 and S6 for SB at pH 7 and 10.8, respectively.

**Figure 1:**
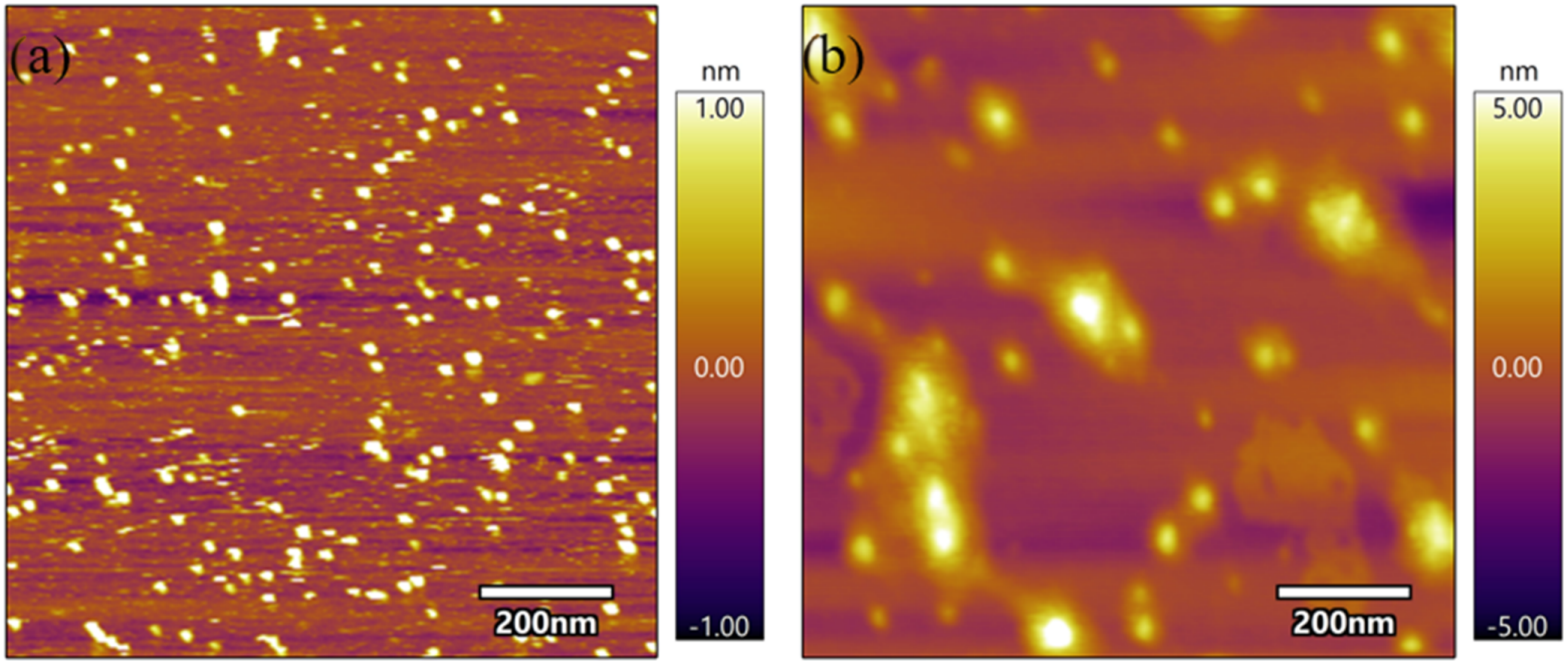
Atomic force microscopy (AFM) images of unheated stem bromelain diluted in (a) pH 7.0 phosphate buffer solution and (b) pH 10.8 borate buffer solution. Scale bar is 200 nm.

After heating the SB solution in pH 10.8 borate buffer for 10 h at 65 °C and incubated at room temperature, it undergoes a conformational change that promotes its self-assembly into amyloid fibrils. Figures 2 and S3 illustrate the fibrils of SB incubated at room temperature for 1, 3, and 7 days, respectively. Small and isolated amyloidogenic growth of fibril structures formed on the first day of incubation, and looking at the magnified image of Figure 2b, finer fibrils were freshly grown. The fibrils typically range from several hundred nanometers to a few micrometers in length, with limited connectivity and alignment. By the third day of incubation, a more pronounced fibrillar network and aggregation become evident, with fibril lengths extending to approximately 2–3 μm, accompanied by increased density and bundling. In Figure 2c,d, a thicker and more defined fibrillar structure dominates the surface of mica substrate. The fibrils have become more extended and connected, suggesting continued aggregation and structural organization over time. A more interconnected network is observed; this is probably due to its growth on the surface of bare mica. In addition to the intrinsic properties of stem bromelain amyloid formation, surface effects may also contribute to the growth architecture. The structures have increased in size where they formed a network on mica substrate. At 7 days, Figures 2f and S3e,f show parallel alignment of amyloid fibril, with lengths reaching several micrometers, often more exceeding 3–5 μm, and exhibiting high structural organization compared to 1- and 3-day incubation. We also performed far-UV circular dichroism (CD) measurements, as shown in Figure S1. The spectra of unheated SB and heated-then incubated SB in pH 10 borate buffer were compared. The unheated SB solution exhibited a spectrum characteristic of an α-helix dominant structure, with distinct negative peaks at 208 and 222 nm. After heating and incubation, these peaks decreased and a single broad minimum appeared, indicating an increase in β-sheet content. These results suggest that the aggregates formed by SB under these conditions possess structural features typical of amyloid fibrils.

**Figure 2:**
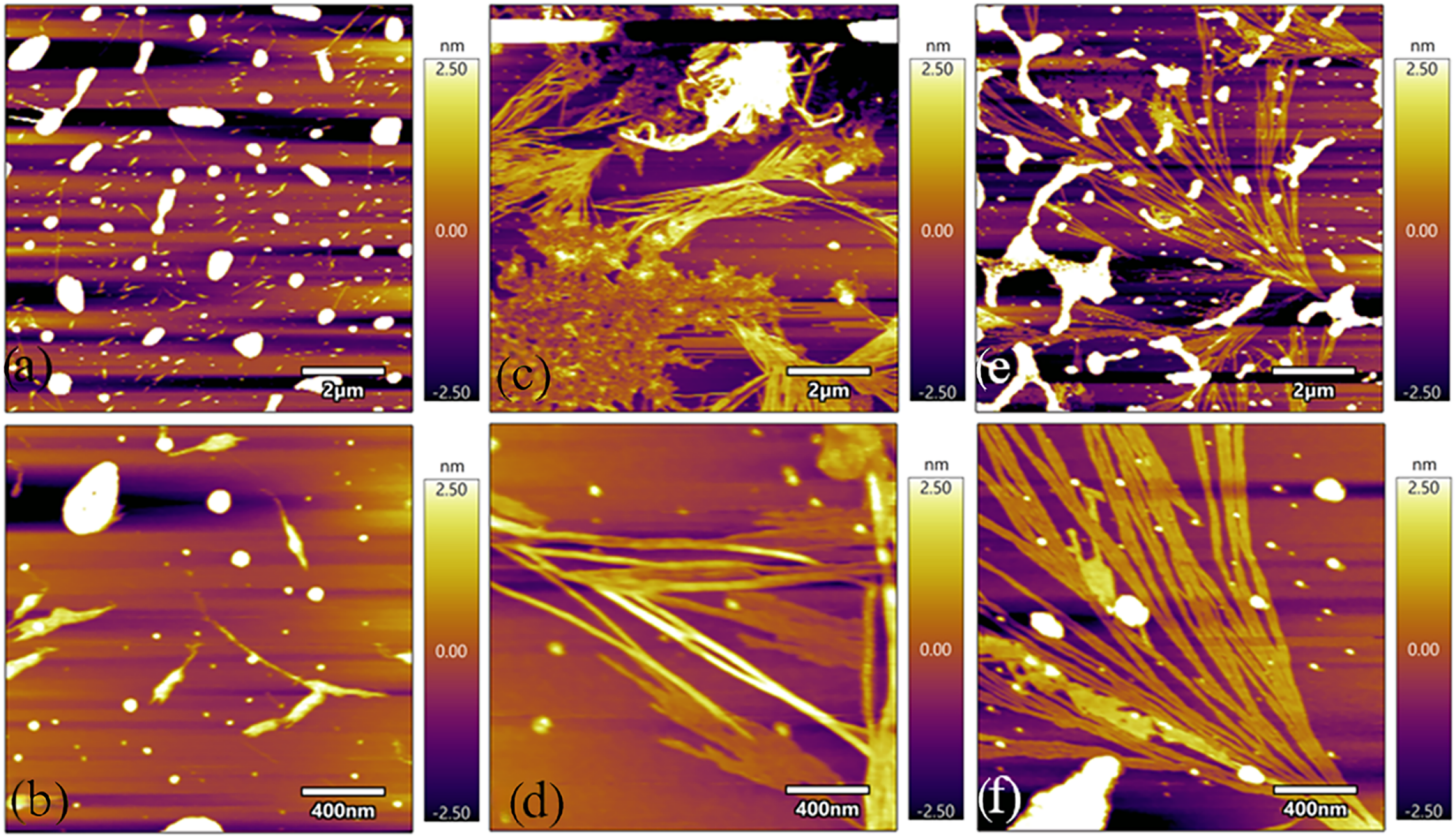
Atomic force microscopy (AFM) time-course images of stem bromelain after incubation and placed on the surface of bare mica substrate, (a, b) 1 day incubation and (c, d) 3 days incubation. (e, f) 7 days incubation.

Quantitative measurements based on the AFM data were analyzed in Figure 3. To provide a more detailed characterization of stem bromelain morphology, analysis using the MATLAB FiberApp application was conducted. We measured the orientation distribution of stem bromelain fibrils at various incubation days. As shown in the representative polar plots from each AFM data (Figure 3a–c), day 1 fibrils exhibited relatively random and dispersed orientations, reflected by the low orientation order parameter (*S*_2D_ = 0.26). By day 3, a moderate increase in alignment was observed (*S*_2D_ = 0.62) and by day 7, the fibrils show a dominant directional alignment with significantly higher *S*_2D_ value (*S*_2D_ = 0.86), indicating the emergence of a localized ordered fibrillar network. This trend is further supported by the normalized fibril orientation distribution (Figure 3d), which was derived by measuring the angles of individual fibrils relative to a defined reference direction (central peak height was set to 0°) from AFM images, while the shaded area represents the standard deviation. Day 1 exhibits a broad distribution with a widespread of angles across the full ±90° range. The fibrils indicate a random and disordered orientation with no strong preference for a particular alignment direction. At day 3, the orientation distribution begins to shift to a narrower peak at 0°, suggesting emergence of preferential alignment compared to day 1. Although there is still significant angular dispersion, some fibrils began to align along dominant direction. Day 7 shows a stiff peak at 0°, which reflects the preferential growth of SB fibrils along the reference direction, with reduced angular dispersion. The relatively small shaded (standard deviation) region also confirms increased uniformity in fibril orientation across multiple AFM images. This reflects a high degree of structural organization and alignment of the fibrils, suggesting that prolonged incubation promotes directional growth and ordering. The observed directional growth of stem bromelain fibrils on the mica substrate represents a novel finding that has not been reported in previous SB studies.

**Figure 3:**
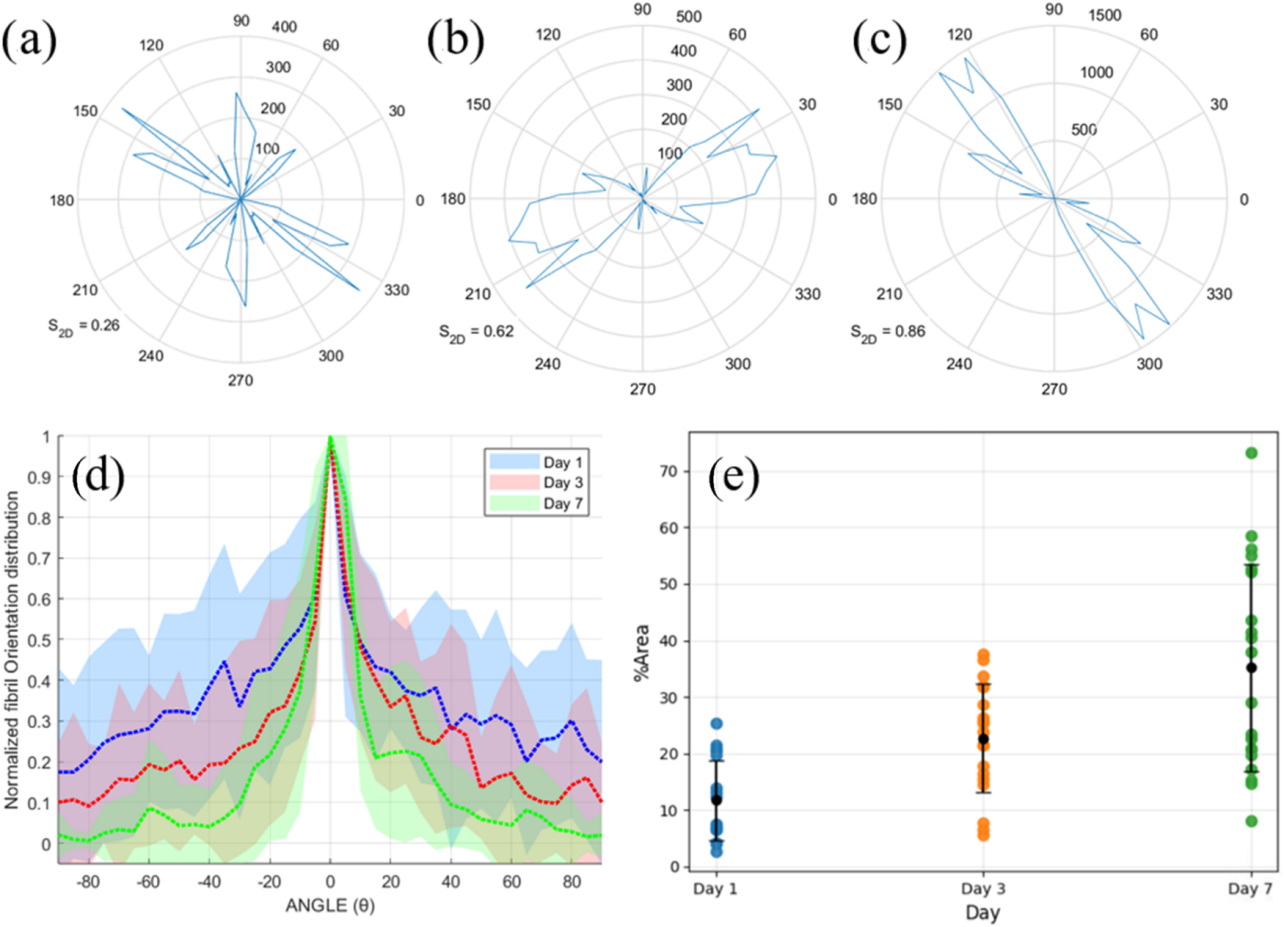
(a–c) Representative polar plots showing the orientational distribution of stem bromelain fibrils at (a) day 1, (b) day 3, and (c) day 7 of incubation. (d) Normalized fibril orientation distribution across day 1, day 3, and day 7. The angle (θ) is set such that 0° corresponds to the dominant fibril alignment direction. Shaded areas represent the standard deviation. (e) Percentage of area occupied by stem bromelain fibrils (coverage area) at each incubation time point, derived from multiple AFM images.

Although surface-induced alignment cannot be ruled out, the observed trend is likely influenced by fibril–fibril interactions within dense networks; therefore, we continued our analysis of the coverage area of SB fibrils. Figure 3e shows the coverage area of stem bromelain fibrils at various incubation days analyzed from the phase retrace images obtained from AFM data. Coverage area refers to the proportion of the mica substrate surface occupied by fibrils, indicating how extensively the fibrils have spread or aggregated across the observed field. An upward trend is clearly observed, which indicates the gradual increase of fibril density over time. Day 1 has mean values of 11.7 ± 7.1, 22.7 ± 9.5, and 35.2 ± 18.4 for 3 and 7 days, respectively. However, day 7 has a notably high fluctuation of values, which means some areas are highly congested with fibrils while some are not. This can also be attributed to the heterogeneous structural organization of amyloid fibrils over time. SB amyloid fibril increased in areas where elongation and organization occur. In Figure S8, the statistical analysis (*t*-test, *p* < 0.05) proved that significant fibrillation growth occurs during the different incubation period.

The molecular aggregation mechanism (nucleation mechanism) of generating amyloid fibrils is a process characterized by the structural transformation of soluble protein monomers into highly ordered fibrillar aggregates. The process begins with primary nucleation, where misfolded monomers aggregate into small oligomeric nuclei. These nuclei then grow through monomer addition (elongation) as individual protein units (monomers) attach to the ends of growing fibrils. As the process advances, secondary nucleation can occur when new fibrils are generated on the surface of existing fibrils, accelerating the overall fibril formation. The fibrils then undergo maturation, forming extensive, often polymorphic, networks of protofibrils and mature fibrils through lateral association and structural rearrangement.^34,35^

We captured the real-time nucleation mechanism of SB protofibril growth on a mica substrate through direct AFM time-lapse imaging, as shown in Figures 4 and S4. The AFM scan rate was 4 min and 16 s. Initially, small nucleation sites emerge, forming bright spots that represent the nucleation stage where monomers aggregate to form unstable oligomers. In this case, the “monomers” refer to the conformationally altered form of stem bromelain under alkaline and heat-treated conditions. Then, they progressively elongate into protofibrils. Over time, these fibrils increase in length, density, and alignment, indicating a directional self-assembly process driven by molecular interactions. The images show a clear progression from isolated fibrils to a more interconnected network, suggesting a continuous monomer addition of SB and connection to already existing fibrils. The amyloid fibrils grown on the mica substrate produced multiple interconnected networks from nucleation sites (secondary nucleation). Moreover, Figure 4k provides a 3D perspective of the fibril growth, revealing a twisted morphology during the early stages—consistent with the structural polymorphism characteristic of amyloid fibrils. Although different formations may also occur in amyloid fibrils, complex polymorphisms can also be observed.

**Figure 4:**
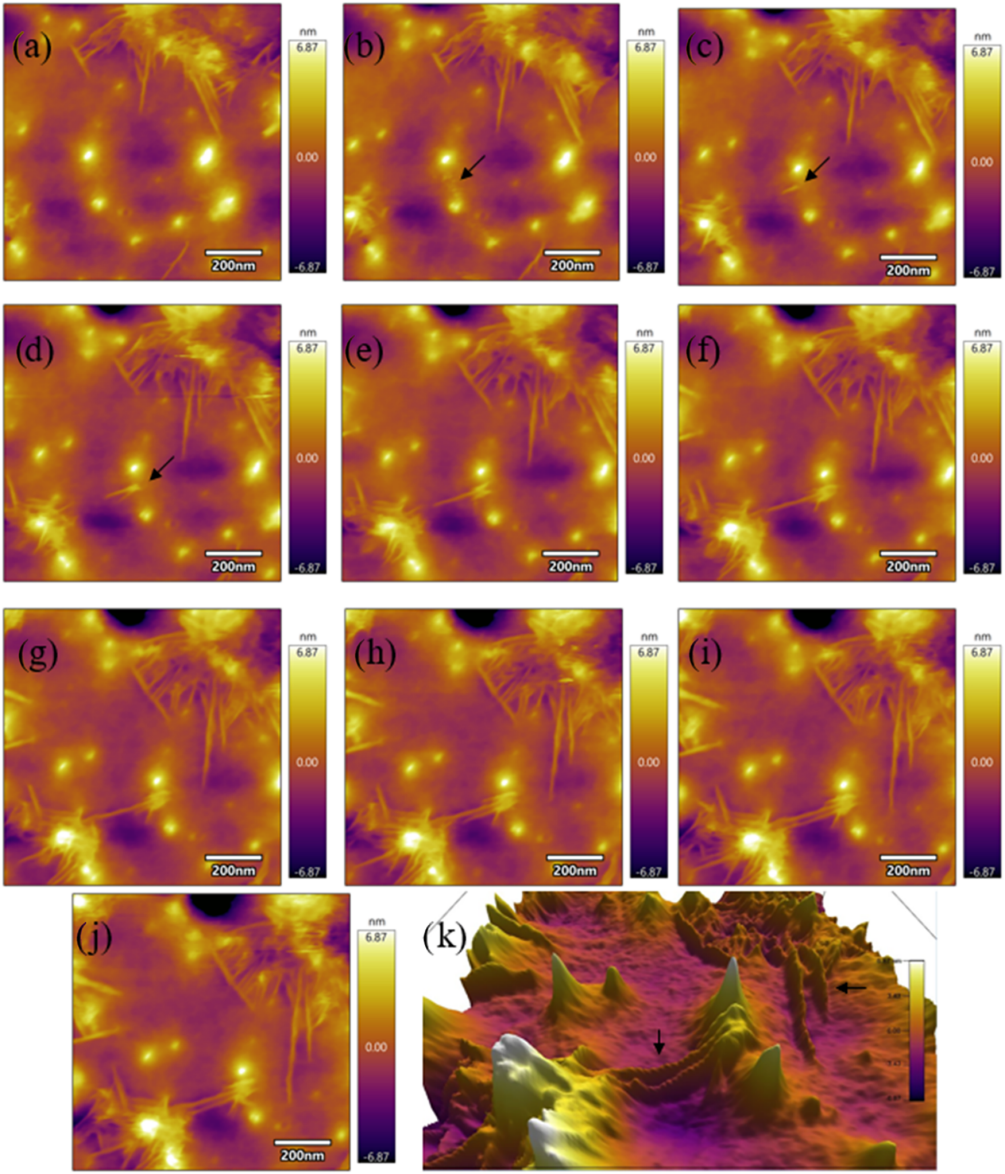
(a–j) Atomic force microscopy (AFM) time-lapse images of the growth of stem bromelain protofibril. (k) 3D perspective view of the freshly grown SB fibril. Scan rate is 4 min and 16 s. Scale bar is 200 nm.

## Conclusions

The research demonstrated that stem bromelain undergoes amyloid fibrillation after heat treatment, with fibrillation continuing to grow during subsequent incubation at room temperature. Time-course AFM imaging revealed a progressive increase in fibril density over time, with a more organized and interconnected network observed on day 7. The orientational distribution analysis showed increasing fibril alignment over time with day 7 exhibiting a dominant directional growth and reduced angular dispersion. The presence of high fluctuations in the fibril coverage area suggests heterogeneous structural organization. The real-time observation of SB protofibrils using AFM in air provided an effective and accessible method for tracking fibrillation dynamics, allowing for easy direct visualization of fibril elongation and network formation. These findings suggest that the continued growth of SB fibrils at room temperature occurs without continuous energy input, indicating minimal requirements for both SB handling and the development of SB-based biomaterials. Furthermore, it highlights the self-assembling nature of stem bromelain amyloid fibrils on mica substrate and demonstrates a technique in AFM in air that provides a practical approach for real-time studies of amyloid fibrillation, providing a practical tool for evaluating aggregation control and formulation strategies.

## Supplementary Material



## Notes

Far-UV circular dichroism (CD) spectra of (blue) unheated stem bromelain (SB), and (orange) heated-then incubated SB in borate buffer solution, percentage of the secondary structure motifs of SB estimated by the BeStSel method for secondary structure determination and fold prediction from the CD spectra; AFM image of pH 10.8 borate buffer solution; other experimental data of the observed stem bromelain fibrils: 1, 3, and 7 days; other experimental data of the sequential AFM time-lapse of the growth of stem bromelain; AFM in liquid observation of stem bromelain particles at pH 7 phosphate buffer solution, room temperature on a bare mica substrate; AFM in liquid observation of stem bromelain particles at pH 10.8 borate buffer solution, room temperature: bare mica substrate, APTES-glutaraldehyde-treated mica substrate; sample AFM phase retrace image of stem bromelain fibril for coverage area measurement, the threshold was adjusted to obtain a black background for mica substrate and white for the samples using ImageJ, sample images of postprocessed stem bromelain fibril samples for coverage area calculation; statistical analysis of fibril coverage area across incubation times using unpaired two-tailed *t*-tests; and AFM calibration using a platinum-coated calibration grid (PDF)

## Abbreviations

AFM: atomic force microscopy
SB: stem bromelain
CFC: closed fluid cell
APTES: (3-aminopropyl) triethoxysilane
CD: circular dichroism

## References

[1] Allison D. P., Mortensen N. P., Sullivan C. J., Doktycz M. J. (2010). Atomic force microscopy
of biological samples. Wiley Interdiscip. Rev.:
Nanomed. Nanobiotechnol..

[2] Chang K.-C., Chiang Y.-W., Yang C.-H., Liou J.-W. (2012). Atomic force microscopy
in biology and biomedicine. Tzu Chi Med. J..

[3] Giessibl F. J. (2005). AFM’s
path to atomic resolution. Mater. Today.

[4] Sumaiya S. A., Liu J., Baykara M. Z. (2022). True Atomic-Resolution Surface Imaging and Manipulation
under Ambient Conditions via Conductive Atomic Force Microscopy. ACS Nano.

[5] Giessibl F. J. (1995). Atomic
Resolution of the Silicon (111)-(7 × 7) Surface by Atomic Force
Microscopy. Science.

[6] Wojdyla M., Raj S., Petrov D. (2012). Absorption
spectroscopy of single red blood cells in
the presence of mechanical deformations induced by optical traps. J. Biomed. Opt..

[7] Adamcik J., Mezzenga R. (2012). Study of amyloid fibrils via atomic
force microscopy. Curr. Opin. Colloid Interface
Sci..

[8] Bergaglio T., Kummer N., Bhattacharya S., Thompson D., Campioni S., Nirmalraj P. N. (2025). On Levodopa
Interactions with Brain Disease Amyloidogenic
Proteins at the Nanoscale. ACS Omega.

[9] Maji S. K., Wang L., Greenwald J., Riek R. (2009). Structure–activity
relationship of amyloid fibrils. FEBS Lett..

[10] Ruggeri F. S., Šneideris T., Vendruscolo M., Knowles T. P. J. (2019). Atomic force
microscopy for single molecule characterisation of protein aggregation. Arch. Biochem. Biophys..

[11] Nirmalraj P. N., Schneider T., Lüder L., Felbecker A. (2023). Protein fibril
length in cerebrospinal fluid is increased in Alzheimer’s disease. Commun. Biol..

[12] Adamcik J., Jung J.-M., Flakowski J., De Los Rios P., Dietler G., Mezzenga R. (2010). Understanding amyloid
aggregation
by statistical analysis of atomic force microscopy images. Nat. Nanotechnol..

[13] Iadanza M. G., Jackson M. P., Hewitt E. W., Ranson N. A., Radford S. E. (2018). A new era
for understanding amyloid structures and disease. Nat. Rev. Mol. Cell Biol..

[14] Yadav S. S., Padhy P. K., Singh A. K., Sharma S., Tanu, Fatima S., Sinha A., Tariq R., Varsha, Sharma S. K., Priya S. (2024). Advancements in amyloid-based
biological materials for healthcare, environmental and sensing applications. Mater. Adv..

[15] Charnley M., Gilbert J., Jones O. G., Reynolds N. P. (2018). Characterization
of Amyloid Fibril Networks by Atomic Force Microscopy. Bio-Protoc..

[16] Carapeto A. P., Marcuello C., Faísca P. F. N., Rodrigues M. S. (2024). Morphological
and Biophysical Study of S100A9 Protein Fibrils by Atomic Force Microscopy
Imaging and Nanomechanical Analysis. Biomolecules.

[17] Han X., He G. (2018). Toward a Rational Design
to Regulate β-Amyloid Fibrillation
for Alzheimer’s Disease Treatment. ACS
Chem. Neurosci..

[18] Makowski L. (2020). The Structural
Basis of Amyloid Strains in Alzheimer’s Disease. ACS Biomater. Sci. Eng..

[19] Hazari M. A., Kannan G., Dasgupta S., Pavan M. K., Jha A. K., Sultana F., Pujahari S. R., Singh S., Dutta S., Pydi S. P., Dutta S., Zafar H., Bhaumik P., Kumar A., Sen S. (2025). Faster Amylin
Aggregation on Fibrillar
Collagen I Hastens Diabetic Progression through β-Cell Death
and Loss of Function. J. Am. Chem. Soc..

[20] Otzen D., Riek R. (2019). Functional amyloids. Cold Spring Harbor Perspect.
Biol..

[21] Fowler D. M., Koulov A. V., Balch W. E., Kelly J. W. (2007). Functional
amyloid--from
bacteria to humans. Trends Biochem. Sci..

[22] Das S., Jacob R. S., Patel K., Singh N., Maji S. K. (2018). Amyloid
Fibrils: Versatile Biomaterials for Cell Adhesion and Tissue Engineering
Applications. Biomacromolecules.

[23] Díaz-Caballero M., Navarro S., Ventura S. (2021). Functionalized Prion-Inspired Amyloids
for Biosensor Applications. Biomacromolecules.

[24] Afjadi, M. N. ; Aziziyan, F. ; Farzam, F. ; Dabirmanesh, B. Biotechnological Applications of Amyloid Fibrils. In Progress in Molecular Biology and Translational Science; Dabirmanesh, B. ; Uversky, V. N. , Eds.; Academic Press, 2024; Chapter 13, Vol. 206, pp 435–472. 10.1016/bs.pmbts.2024.04.001 38811087

[25] Zaman M., Ehtram A., Chaturvedi S. K., Nusrat S., Khan R. H. (2016). Amyloidogenic
behavior of different intermediate state of stem bromelain: A biophysical
insight. Int. J. Biol. Macromol..

[26] Zaman M., Mohammad Z. S., Saima N., Vahid K. M., Atiyatul Q., Rehan A. M., Khan R. H. (2017). Surfactant-mediated
amyloidogenesis
behavior of stem bromelain; a biophysical insight. J. Biomol. Struct. Dyn..

[27] Pavan R., Jain S., Shraddha, Kumar A. (2012). Properties and therapeutic
application
of bromelain: a review. Biotechnol. Res. Int..

[28] Murachi T., Yamazaki M. (1970). Changes in conformation
and enzymic activity of stem
bromelain in alkaline media. Biochemistry.

[29] Dave S., Mahajan S., Chandra V., Dkhar H. K., Sambhavi, Gupta P. (2010). Specific molten globule
conformation of stem bromelain at alkaline pH. Arch. Biochem. Biophys..

[30] Ramli A. N. M., Manas N. H. A., Hamid A. A. A., Hamid H. A., Illias R. M. (2018). Comparative
structural analysis of fruit and stem bromelain from *Ananas comosus*. Food Chem..

[31] Kardos J., Nyiri M. P., Moussong É., Wien F., Molnár T., Murvai N., Tóth V., Vadászi H., Kun J., Jamme F., Micsonai A. (2025). Guide to the
structural characterization
of protein aggregates and amyloid fibrils by CD spectroscopy. Protein Sci..

[32] Usov I., Mezzenga R. (2015). FiberApp: An Open-Source Software for Tracking and
Analyzing Polymers, Filaments, Biomacromolecules, and Fibrous Objects. Macromolecules.

[33] Hart P. E., Nilsson N. J., Raphael B. (1968). A Formal Basis
for the Heuristic
Determination of Minimum Cost Paths. IEEE Trans.
Syst. Sci. Cybern..

[34] Michaels T. C., Šarić A., Habchi J., Chia S., Meisl G., Vendruscolo M., Dobson C. M., Knowles T. P. (2018). Chemical
kinetics
for bridging molecular mechanisms and macroscopic measurements of
amyloid fibril formation. Annu. Rev. Phys. Chem..

[35] Knowles T. P. J., Vendruscolo M., Dobson C. M. (2014). The amyloid state and its association
with protein misfolding diseases. Nat. Rev.
Mol. Cell Biol..

