# Supplementary material for: Amyloidogenic Growth Observation of Stem Bromelain via Atomic Force Microscopy
Source: ACS Omega. 2025 Oct 9;10(41):49002–9. doi: 10.1021/acsomega.5c07595 (PMC12547772; doi:10.1021/acsomega.5c07595)
Supplement: Supplementary file 1 [file ao5c07595_si_001.pdf]

Supporting Information

Amyloidogenic growth observation of stem bromelain via atomic force microscopy

Maria Christine Lugo[1], Atsushi Kanmura[1], Toshiharu Kobayashi[1], Masahiro Ito[2], Takunori Harada[3], Kazuo Umemura[1]*

[1]Department of Physics, Tokyo University of Science, 1-3 Kagurazaka, Shinjuku, Tokyo 162-8601, Japan

[2] Department of Medical Course, Teikyo Heisei University, 2-51-4 Higashi-ikebukuro, Toshima, Tokyo 1708445, Japan

[3] Department of Integrated Science and Technology, Faculty of Science and Technology, Oita University, 700 Dannoharu, Oita City 870-1192, Japan

*Corresponding author: Maria Christine Lugo-1223705@ed.tus.ac.jp

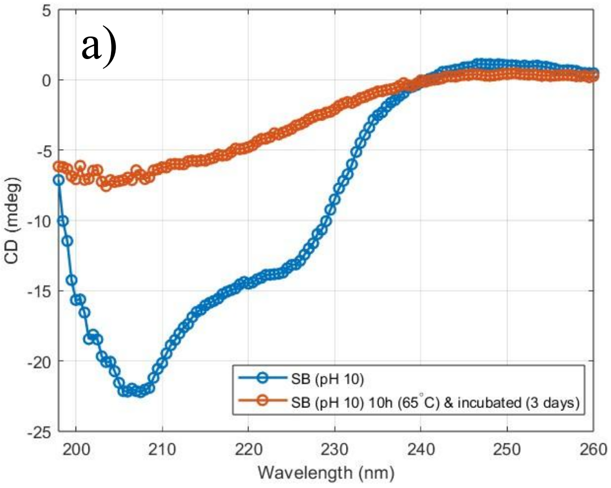

b)

Secondary structures of SB

	BESTSEL	
	Unheated SB	Heated and incubated SB
**Helix**	1.5	0.0
**Beta**	35.2	35.9
**Turn**	16.1	16.3
**Random**	47.2	47.8
**RMSD**	0.0201	0.0223

Figure S1. a) Far-UV circular dichroism (CD) spectra of (blue) unheated stem bromelain (SB), and (orange) heated then incubated SB in borate buffer solution. b) Percentage of the secondary structure motifs of SB estimated by the BeStSel method for secondary structure determination and fold prediction from the CD spectra.

We further analyzed the stem bromelain solutions using Far－UV circular dichroism (CD). The far-UV CD spectra of stem bromelain (SB) revealed distinct structural changes upon heating and incubation. As shown in Figure a), unheated SB exhibited characteristic negative peaks at 208 nm and 222 nm, indicative of α-helical structures. In contrast, the spectrum of heated and incubated SB displayed a broader profile with a marked reduction in α-helical features and an increased contribution from β-structures. This observation is further supported by the secondary structure analysis using the BeStSel method (Figure b), which showed a decrease in α-helix content from 1.5% to 0.0%, accompanied by an increase in β-structure from 35.2% to 35.9%.

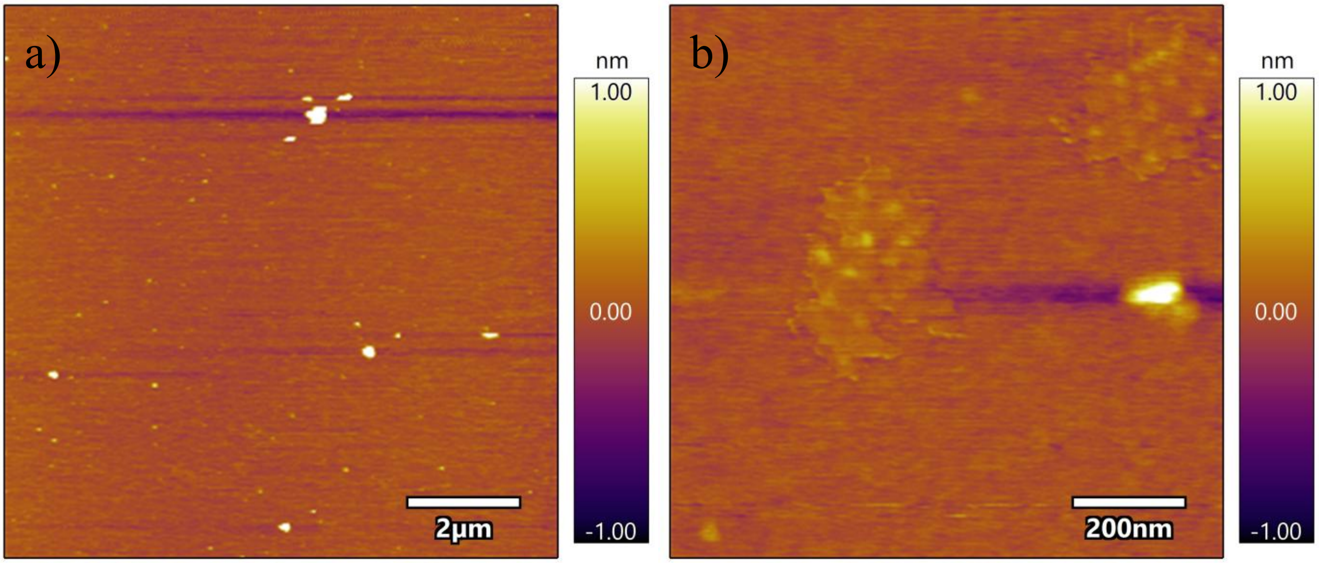

Figure S2. AFM in air images of 10 ul of 100 mM borate buffer solution deposited on the bare mica for 10 minutes followed by three washes with ultrapure water. lone, serving as a control reference for comparison with stem bromelain-treated samples. These representative images confirm minimal salt residue formation from the borate buffer.

As a control, AFM in-air imaging was performed using borate buffer solution without stem bromelain. The larger scan area (Figure a) revealed minimal and scattered particulate deposits across the mica substrate, while the higher magnification image (Figure b) provided clearer visualization of these salt residues. These features are attributed to residual salts originating from the borate buffer. Importantly, no fibrillar structures were detected under these conditions, confirming that any fibril formation observed in subsequent experiments arises specifically from stem bromelain solutions.

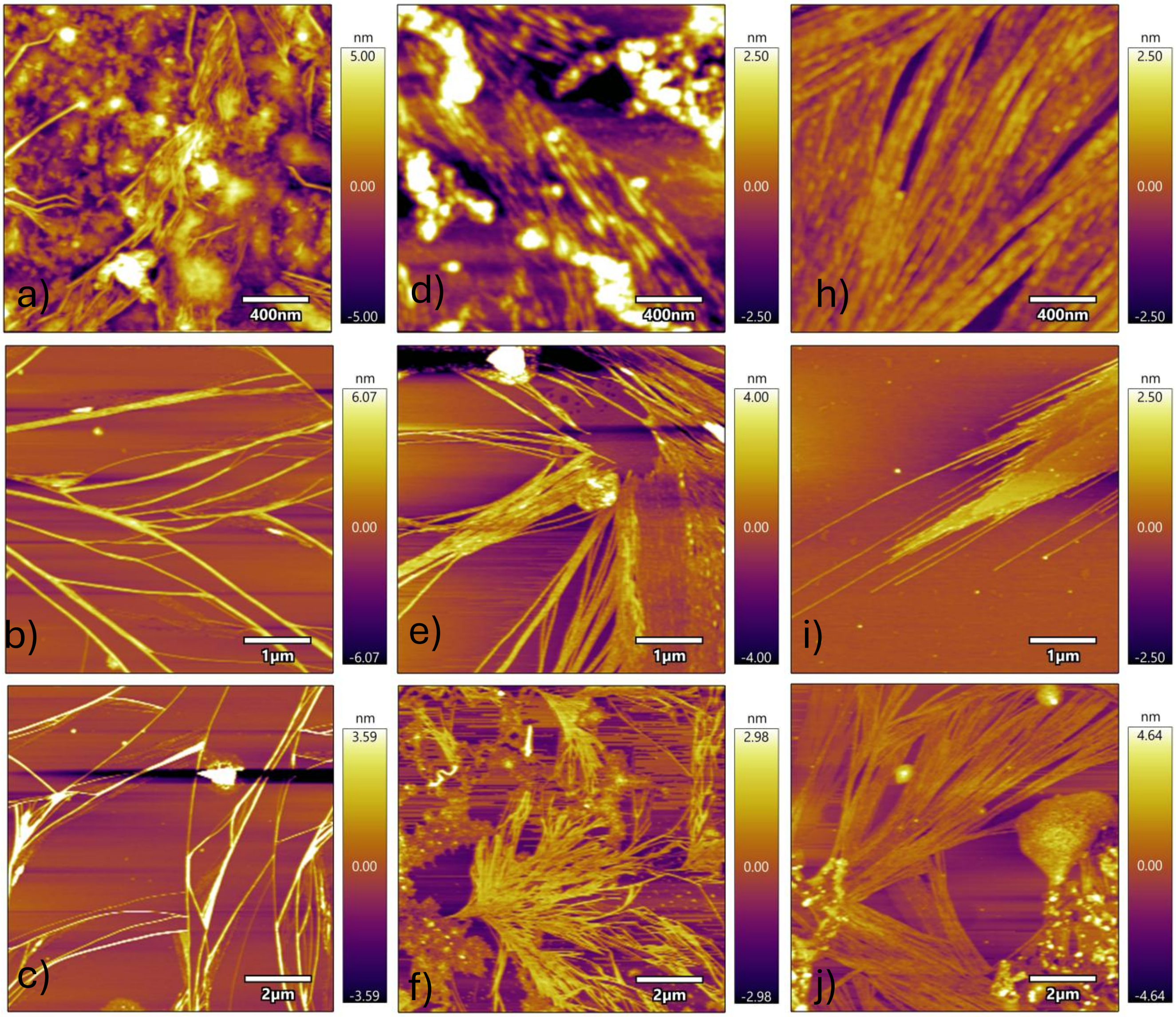

Figure S3. Other experimental data of the observed stem bromelain fibrils a-c) 1 day, d-f) 3 days h-j) 7 days. Scale bar are set to 400 nm, 1 µm, and 2 µm, respectively.

Additional AFM in air images of stem bromelain fibrils for 1, 3 and 7 days of incubation. On Day 1 (a–c), fibrils appear dispersed, and entangled, with limited alignment and lower overall density. By Day 3 (d–f), fibrils become more defined and begin forming localized bundles with increasing length and partial orientation. By Day 7 (h–j), extensive fibril networks are observed, with clear evidence of parallel growth and alignment, indicating the progression of organized fibrillation on mica substrate. Fibril density and bundling increase markedly over time, suggesting ongoing self-assembly and maturation of the fibrillar structures.

Supporting Information 3
Lugo et. al

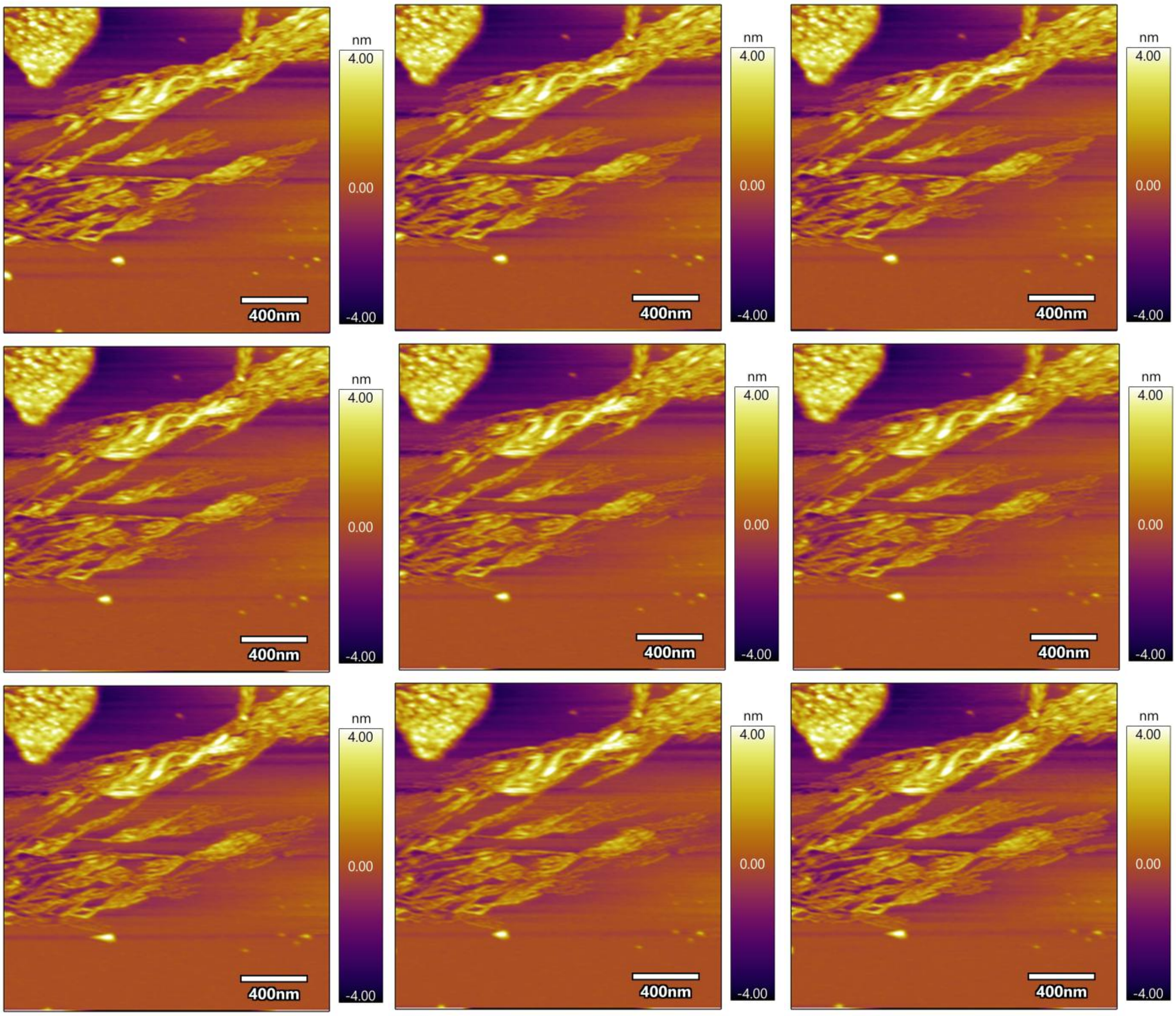

Figure S4. Other experimental data of the sequential AFM time-lapse of the growth of stem bromelain fibrils. Scale bar is set to 400 nm across all images.

The sequential AFM images provide additional evidence of the dynamic growth process of stem bromelain fibrils. Over time, the fibrils can be seen extending in length while also associating laterally with neighboring structures. This progressive elongation and bundling behavior suggests an active self-assembly process that drives the formation of larger fibrillar networks. Such nanoscale organization reflects not only the ability of stem bromelain molecules to form stable fibrillar structures

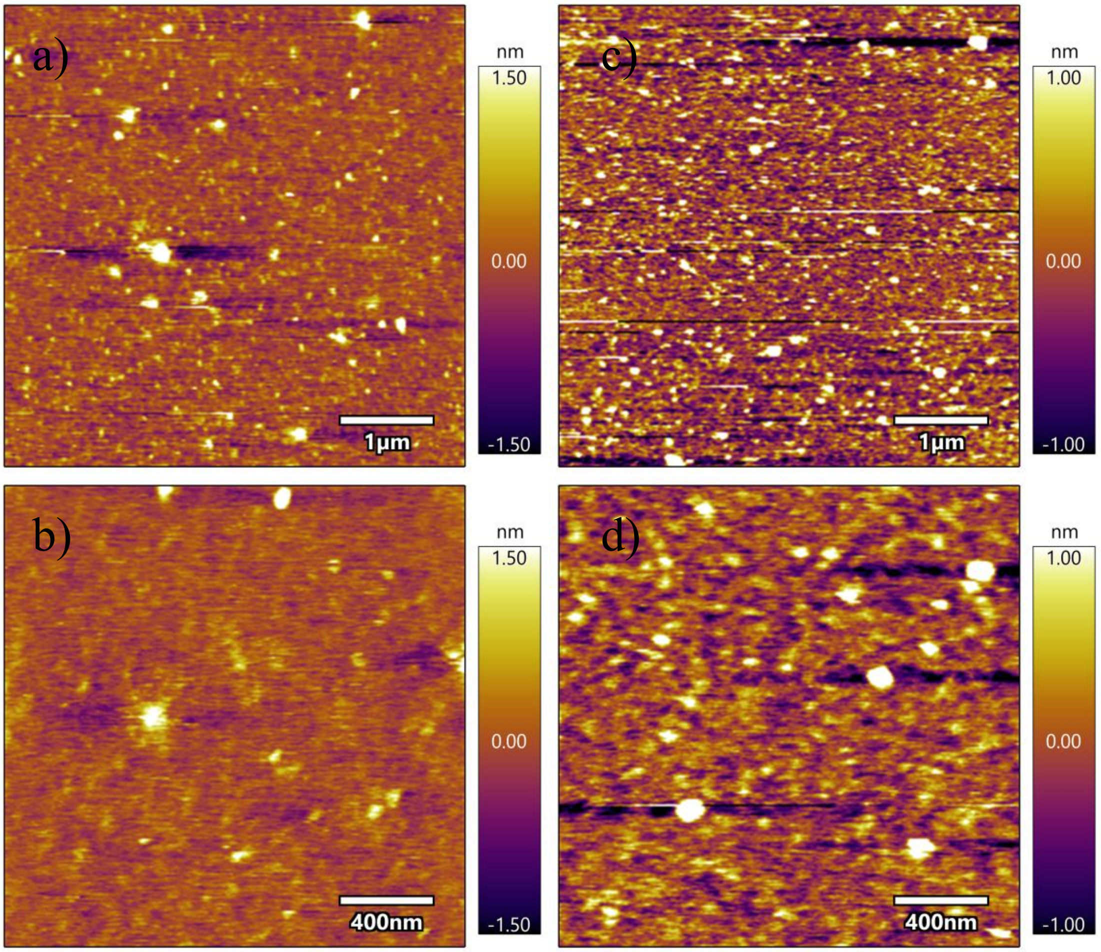

Figure S5. AFM in liquid observation of stem bromelain particles at pH 7 phosphate buffer solution, room temperature on a bare mica substrate

For deposition on bare mica substrate for liquid observation, prepared sample solution was dropped onto a clean parafilm and covered by the bare mica substrate (approx. 1mm by 1mm) for 10 mins. This technique was done to prevent the prepared sample from spilling outside of mica. The substrate was then washed with 100 ul ultrapure water, three times. The prepared substrates were glued to the bottom of a closed fluid cell (CFC) (939.010, Asylum Research, CA, USA) and soaked in 2000 ul buffer solution. AFM in liquid (AC mode, MFP-3D, Asylum Research, CA, USA) measurement was conducted at room temperature. The scan rate was fixed to 1 Hz and scan time of 4 mins and 16 sec.

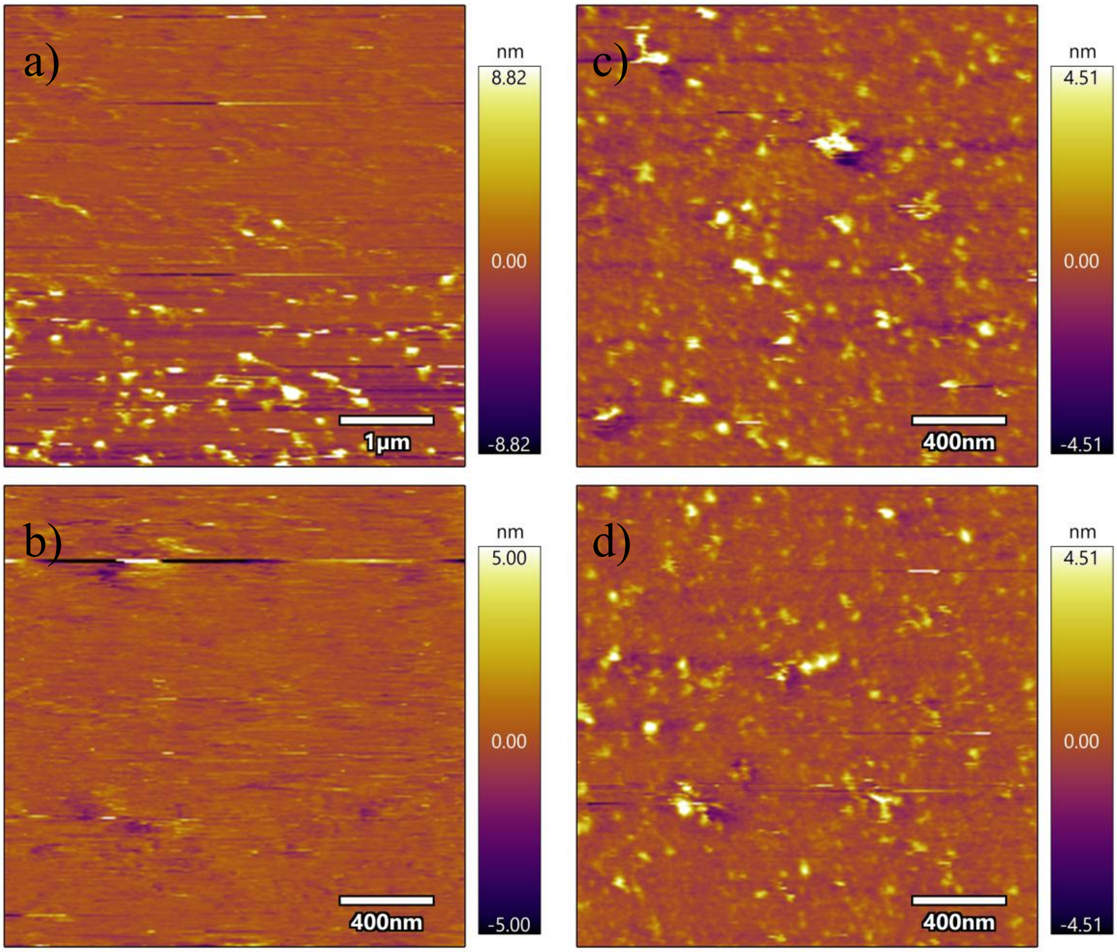

Figure S6. AFM in liquid observation of stem bromelain particles at pH 10.8 borate buffer solution, room temperature. a,b) bare mica substrate, c,d) APTES-glutaraldehyde treated mica substrate

For deposition on (3-Aminopropyl)triethoxysilane (APTES)-glutaraldehyde-treated mica, 10 ul of prepared sample was directly dropped on 0.1% APTES pretreated mica substrate and waited for 10 mins. The substrate was then washed with 100 ul of ultrapure water, three times. Then, 0.5% glutaraldehyde was prepared in a 100mM borate buffer solution and 50 ul of it was dropped on the substrate for 10 mins. Again, it was washed with 100 ul ultrapure water, three times.

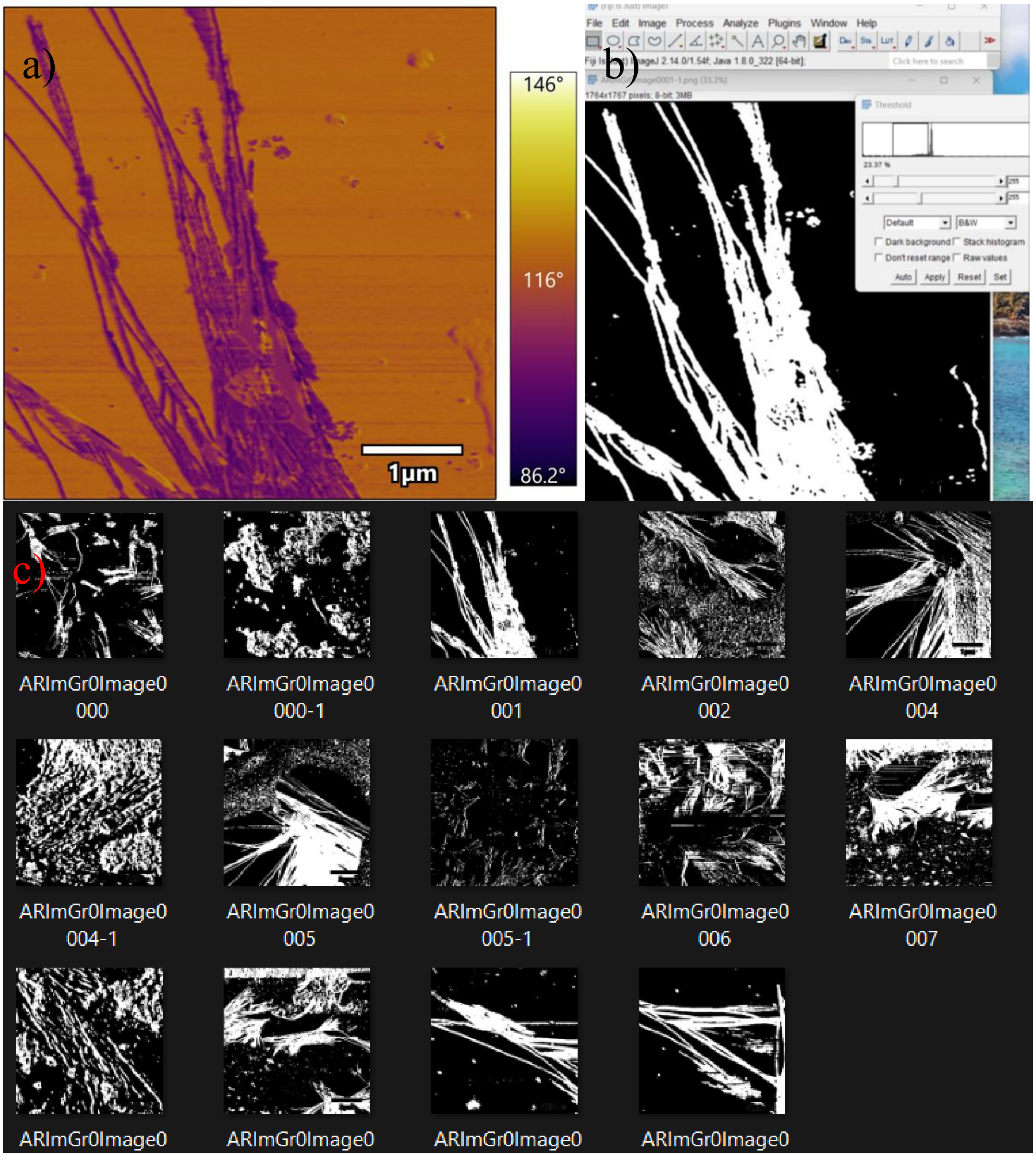

Figure S7. a) Sample AFM phase retrace image of stem bromelain fibril for coverage area measurement, b) the threshold was adjusted to obtain a black background for mica substrate and white for the samples using ImageJ, c) Sample images of post-processed stem bromelain fibril samples for coverage area calculation.

Post-processing of the phase retrace AFM images was performed using ImageJ. Threshold adjustments were set to around 125 for lower, and 255 for upper threshold level, to isolate brighter features (like fibrils) and removes the dimmer background. Quantitative analysis was carried out by calculating the fibril coverage area, defined as the ratio of the total fibrillar area to the overall image area (in pixels). Subsequent statistical analyses were conducted to evaluate the data.

Comparison	Mean (Group A)	Mean (Group B)	t-Stat	p-value (two-tail)	Significant? (α = 0.05)
Day 1 vs Day 3	12.12	23.15	-4.02	0.000317	✅ Yes
Day 1 vs Day 7	12.12	36.11	-5.06	0.0000401	✅ Yes
Day 3 vs Day 7	23.15	36.11	-2.76	0.01016	✅ Yes

Figure S8. Statistical analysis of fibril coverage area across incubation times using unpaired two-tailed *t*-tests. The comparisons show a significant increase in mean area density from Day 1 to Day 3, and from Day 1 to Day 7, indicating continued fibril growth. All comparisons yielded p-values below the significance threshold ($\alpha = 0.05$), confirming statistically meaningful increases in fibril coverage as incubation progresses.

The statistical analysis further supports the time-dependent increase in fibril coverage observed in the AFM images. Between Day 1 and Day 3, the mean coverage area nearly doubled (12.12 vs. 23.15), yielding a *t*-statistic of –4.02 and a highly significant *p*-value of 0.000317. A more pronounced increase was detected when comparing Day 1 and Day 7 (12.12 vs. 36.11), with a *t*-statistic of –5.06 and a similarly significant *p*-value of 0.0000401. Even at later stages, a statistically significant difference persisted between Day 3 and Day 7 (23.15 vs. 36.11; $t = –2.76$, $p = 0.01016$). Since all comparisons resulted in *p*-values below the significance threshold ($\alpha = 0.05$), these findings confirm that fibril formation proceeds progressively over the incubation period, with substantial growth occurring both in the early and later stages.

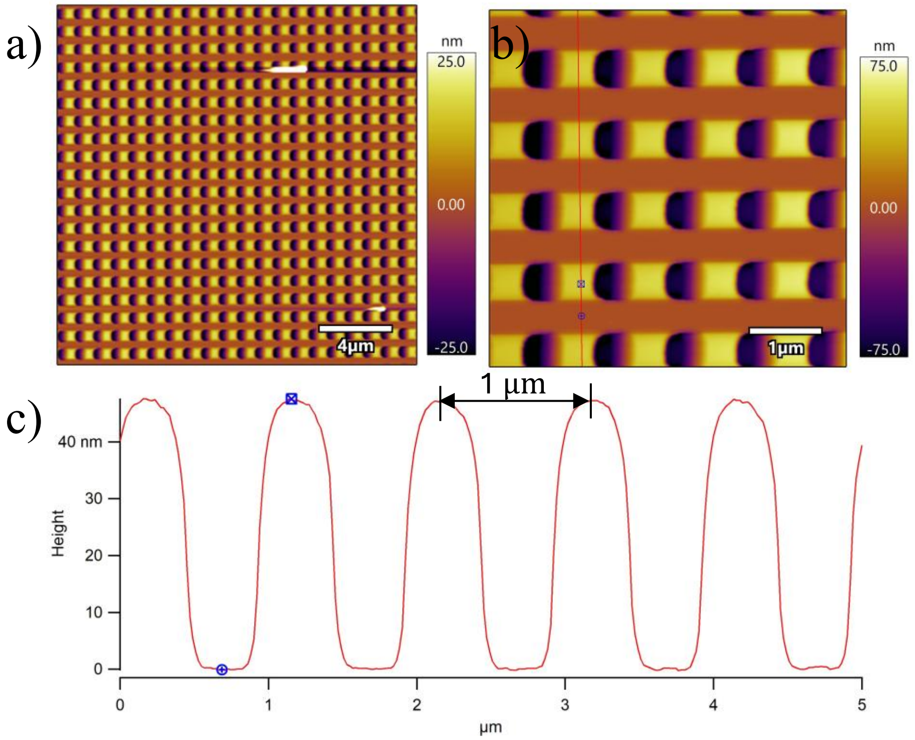

Figure S9. AFM calibration using a platinum-coated calibration grid. (a) Low-magnification AFM image showing the grid structure at a 20 µm scan size. (b) Higher magnification at 5 µm scan length highlights the periodic ridges used for dimensional accuracy. (c) Corresponding height profile extracted from the grid, showing a consistent peak-to-peak spacing of approximately 1 µm, which matches the manufacturer's specifications.

To verify the accuracy of the AFM cantilever, calibration was performed using a manufactured calibration grid (Digital Instruments, Veeco Metrology Group) with a periodicity of 1 µm × 1 µm. Well-defined and consistent periodic ridges were observed in both the 20 µm scan (Figure S9a) and the 5 µm scan (Figure S9b). The corresponding height profiles further confirmed a uniform peak-to-peak spacing of approximately 1 µm, in agreement with the nominal grid dimensions.
